# Accelerated FEV_1_ decline and risk of cardiovascular disease and mortality in a primary care population of COPD patients

**DOI:** 10.1183/13993003.00918-2020

**Published:** 2021-03-04

**Authors:** Hannah R. Whittaker, Chloe Bloom, Ann Morgan, Deborah Jarvis, Steven J. Kiddle, Jennifer K. Quint

**Affiliations:** 1 Respiratory Epidemiology, Occupational Medicine and Public Health, National Heart and Lung Institute, Imperial College London, London, UK; 2 MRC Biostatistics Unit, University of Cambridge, Cambridge, UK; 3 Joint last authors

## Abstract

Accelerated lung function decline has been associated with increased risk of cardiovascular disease (CVD) in a general population, but little is known about this association in chronic obstructive pulmonary disease (COPD). We investigated the association between accelerated lung function decline and CVD outcomes and mortality in a primary care COPD population.

COPD patients without a history of CVD were identified in the Clinical Practice Research Datalink (CPRD)-GOLD primary care dataset (n=36 382). Accelerated decline in forced expiratory volume in 1 s (FEV_1_) was defined using the fastest quartile of the COPD population's decline. A Cox regression was used to assess the association between baseline accelerated FEV_1_ decline and a composite CVD outcome over follow-up (myocardial infarction, ischaemic stroke, heart failure, atrial fibrillation, coronary artery disease and CVD mortality). The model was adjusted for age, sex, smoking status, body mass index, history of asthma, hypertension, diabetes, statin use, Modified Medical Research Council (mMRC) dyspnoea score, exacerbation frequency and baseline FEV_1_ % predicted.

6110 COPD patients (16.8%) had a CVD event during follow-up; median length of follow-up was 3.6 years (interquartile range (IQR) 1.7–6.1 years). Median rate of FEV_1_ decline was –19.4 mL·year^−1^ (IQR –40.5–1.9); 9095 patients (25%) had accelerated FEV_1_ decline (> –40.5 mL·year^−1^), 27 287 (75%) did not (≤ –40.5 mL·year^−1^). Risk of CVD and mortality was similar between patients with and without accelerated FEV_1_ decline (HR_adj_ 0.98, 95% CI 0.90–1.06). Corresponding risk estimates were 0.99 (95% CI 0.83–1.20) for heart failure, 0.89 (95% CI 0.70–1.12) for myocardial infarction, 1.01 (95% CI 0.82–1.23) for stroke, 0.97 (95% CI 0.81–1.15) for atrial fibrillation, 1.02 (95% CI 0.87–1.19) for coronary artery disease and 0.94 (95% CI 0.71–1.25) for CVD mortality. Rather, risk of CVD was associated with a mMRC score ≤2 and two or more exacerbations in the year prior.

CVD outcomes and mortality were associated with exacerbation frequency and severity and increased mMRC dyspnoea score but not with accelerated FEV_1_ decline.

## Abstract 2

**In a primary care population of COPD patients, CVD outcomes and mortality were not associated with accelerated FEV_1_ decline but with frequent and severe exacerbations of COPD and increased breathlessness** https://bit.ly/35APXL6

## Introduction

Forced expiratory volume in 1 s (FEV_1_) declines with age from early adulthood. A previous meta-analysis found that in the general population the average rate of FEV_1_ decline in ageing adults ranged from –9.9 mL·year^−1^ to –56.0 mL·year^−1^ with a median decline of –29.4 mL·year^−1^ [1]. Patients with chronic obstructive pulmonary disease (COPD), however, lose lung function at an accelerated rate, at about –33.2 mL·year^−1^ according to Vestbo *et al*. [2]. The rate of decline in COPD is highly heterogeneous, with ∼38% of patients declining by > –40 mL·year^−1^, 31% at rates between –21 mL·year^−1^ and –40 mL·year^−1^, and 31% by < –21 mL·year^−1^ [2]. Several factors have been associated with the rate of change in lung function in COPD patients, including frequency and severity of acute exacerbations of COPD (AECOPD), smoking and COPD severity [3–8]. However, little is known about the association between the rate of lung function decline and comorbidity in COPD patients.

One of the most prevalent comorbidities in COPD patients is cardiovascular disease (CVD) [9]. Both COPD and CVD share common risk factors, *e.g.* smoking and ageing [10, 11]. Specifically, exposure to toxic particles in cigarette smoke can cause the increased systemic inflammation that characterises both COPD and CVD [12, 13]. It is not fully understood how COPD and CVD are linked beyond their shared risk factors but researchers have identified a number of possible mechanisms, such as hypoxia and oxidative stress, that might be involved [9, 10]. Furthermore, numerous studies have reported associations between various measures of impaired lung function, including low FEV_1_, forced vital capacity (FVC) and FEV_1_/FVC, and an increased likelihood of developing CVD, as well as an increased risk of hospitalisation and death secondary to CVD [14–20].

More recently, it has been suggested that the rate at which lung function is lost is associated with risk of CVD. In a general population study of participants in the Atherosclerosis Risk in Communities (ARIC) study, accelerated decline in FEV_1_ over a baseline period of 3 years was associated with an increased risk of hospitalisation and death from heart failure and stroke [21]. To date, no studies have investigated the association between rate of FEV_1_ decline and risk of CVD outcomes and mortality in patients with COPD, who are already at greater risk of CVD than the general population [9]. We therefore investigated whether COPD patients with accelerated FEV_1_ decline were more likely to develop CVD in a primary care population of COPD patients in England.

## Methods

### Study population and design

Clinical Practice Research Datalink (CPRD)-GOLD is a primary care electronic healthcare record database. It contains information on general practitioner (GP) practices in the UK, including consultations, patient demographics, therapies prescribed and clinical diagnoses. CPRD contains ∼7% of the UK's population and is generalisable in terms of age, sex and ethnicity [22]. Linked pseudonymised data from Hospital Episode Statistics (HES) and the Office of National Statistics (ONS) were provided for this study by CPRD for patients in England. Data were linked by NHS Digital using identifiable data held only by NHS Digital. General practices consent to this process at a practice level, with individual patients having the right to opt-out. HES and ONS data were used to identify CVD hospitalisations and deaths.

**FIGURE 1:**
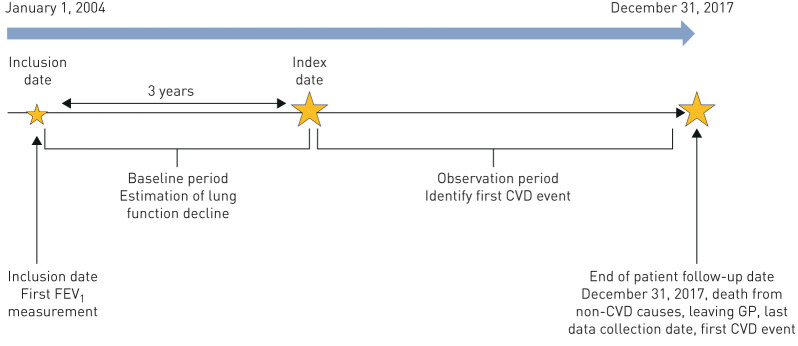
Study design. FEV_1_: forced expiratory volume in 1 s; CVD: cardiovascular disease; GP: general practitioner.

Patients were included if they met the following minimum inclusion criteria: patients were 1) eligible for HES linkage, 2) diagnosed with COPD, 3) aged 35 years or older, 4) current or ex-smokers and 5) had data recorded from 2004 onwards. Specifically, the inclusion date was the date of the patients' first FEV_1_ measurement after the date on which they were diagnosed with COPD, registered with their current GP, aged 35 years, and the date at which the practice was deemed of research quality (figure 1) [22].

Following the inclusion date, patients were required to have 3 years of baseline follow-up with at least two FEV_1_ measurements at least 6 months apart in order to estimate the patient's rate of FEV_1_ decline; changes in lung function should be estimated over longer periods to draw conclusions about long-term lung function decline and to reduce possible measurement error [23]. The index date was the date at the end of the baseline period and indicated the start of follow-up (figure 1). Patients were consequently followed up from their index date until December 31, 2017, or the first date of any of the following events: transferred to a non-CPRD GP practice, the last data collection date, died from non-CVD causes or had a CVD event. In addition, patients were required to have no history of stroke, heart failure, myocardial infarction, atrial fibrillation or coronary artery disease ever recorded prior to the index date.

### COPD and FEV_1_ decline

Patients with COPD were identified using a validated algorithm for COPD in CPRD-GOLD [24]. Patients aged 35 years and over who had at least one record denoting a clinical diagnosis of COPD and a history of smoking (ex or current) were considered to have COPD. Never-smokers were excluded owing to the potential misclassification of COPD with asthma. The exposure of interest was accelerated FEV_1_ decline. For each study-eligible patient, all absolute FEV_1_ measurements recorded in CPRD-GOLD between the inclusion date and the index date were identified and the rate of FEV_1_ decline estimated using mixed linear regression modelling with random intercepts and random slopes. Accelerated decline was defined as patients whose FEV_1_ decline fell in the fastest quartile of the decline. Patients were classed as either with or without accelerated FEV_1_ decline. In a recent validation study, the quality of spirometry measurements was found to be high [25].

### CVD outcomes

The primary outcome was a composite measure defined as the time to first CVD event during follow-up. A CVD event comprised myocardial infarction, heart failure, stroke, atrial fibrillation, coronary artery disease excluding acute myocardial infarction, and CVD mortality. These events were identified through primary care records (CPRD-GOLD), hospital admissions data (HES) and mortality statistics (ONS). International Classification of Diseases, 10th revision (ICD-10) codes were used to identify hospitalisations (primary diagnosis) and CVD deaths. CVD events that were recorded on the same day in CPRD-GOLD, HES and/or ONS were further explored to avoid duplication of events. In these cases, mortality events were prioritised, followed by hospitalisations and then GP-recorded events. Secondary outcomes included time to first myocardial infarction, heart failure, stroke, atrial fibrillation, coronary artery disease or CVD mortality.

### Statistical analyses

Baseline characteristics were described using proportions, medians and interquartile ranges (IQR). Cox regression was used to investigate time to the first composite CVD event, comparing patients with and without accelerated FEV_1_ decline, adjusted for sex, age (continuous), smoking status (current or ex-smoker) and level of airflow obstruction (mild: FEV_1_ ≤80% predicted; moderate: FEV_1_ 50%–80% predicted; severe: FEV_1_ 30%–50% predicted; very severe: FEV_1_ <30% predicted; based on patients' last baseline FEV_1_ measurement, height and sex), which were identified at index date. Modified Medical Research Council (mMRC) dyspnoea score (0–4) and body mass index (BMI) (underweight, normal, overweight, obese) were defined as the closest measurement to the index date within the baseline period of 3 years. A history of hypertension, diabetes or asthma was identified within the baseline period of 3 years. Statin use was defined as at least one prescription in the year prior to the index date. AECOPD frequency and severity (moderate: GP-recorded AECOPD; severe: hospitalisation for AECOPD) were also defined using information recorded in the year prior to the index date and categorised as none; one moderate and zero severe; two moderate and zero severe; three or more moderate and zero severe; one severe and any number of moderate; and two or more severe and any number of moderate. This categorisation was similar to that used in previous investigations of AECOPD events in CPRD-GOLD [26]. Secondary analyses investigated the association between accelerated FEV_1_ decline and each separate CVD event, *i.e.* myocardial infarction, heart failure, stroke, atrial fibrillation, coronary artery disease and CVD mortality.

The following sensitivity analyses were also performed: 1) rates of relative change in FEV_1_ and change in FEV_1_ % predicted were used to categorise accelerated decline (as opposed to absolute FEV_1_); 2) risk of CVD outcomes and mortality was compared between patients with accelerated FEV_1_ decline and patients in the other three quartiles separately; 3) the linear relationship between rate of FEV_1_ decline and risk of CVD was investigated; 4) different cut-offs were used (> −20 mL·year^−1^ (reference group), −20­– −40 mL·year^−1^, −40– −60 mL·year^−1^ and < −60 mL·year^−1^) to define four groups of rates of FEV_1_ decline; 5) spline regression was used to assess non-linear relationships between accelerated FEV_1_ decline and risk of CVD outcomes using 10th, 50th and 90th percentiles of FEV_1_ decline; 6) patients with a history of asthma were excluded in case of misclassification of COPD; 7) only hospitalised CVD events were investigated as the outcome; 8) only GP-recorded CVD outcomes were investigated as the outcome; 9) only deaths from CVD were investigated as the outcome; 10) models were stratified by sex, age, smoking status, AECOPD frequency and airflow obstruction (*i.e.* baseline FEV_1_ % predicted); 11) accelerated FEV_1_ decline was categorised using a rate of FEV_1_ decline that was estimated using at least four FEV_1_ measurements during the baseline period; 12) accelerated FEV_1_ decline was categorised using a rate of FEV_1_ decline that was estimated using at least two FEV_1_ measurements at least 2 years apart; 13) the association between accelerated FEV_1_ decline and risk of composite CVD, heart failure, myocardial infarction, stroke, atrial fibrillation and coronary artery disease in the first year of follow-up; and 14) all CVD events during follow-up were identified (not first event only) and Poisson regression was used to model repeated events. All analyses were performed using STATA v16.0 (StatCorp, College Station, TX, USA).

## Results

### Patient characteristics

**FIGURE 2:**
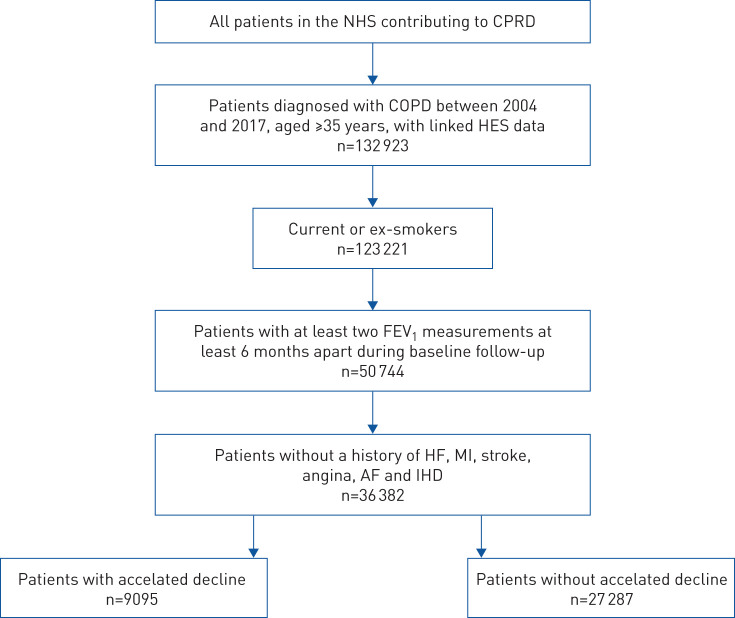
Patients meeting inclusion criteria. NHS: National Health Service; CPRD: Clinical Practice Research Datalink; COPD: chronic obstructive pulmonary disease; HES: Hospital Episode Statistics; FEV_1_: forced expiratory volume in 1 s; HF: heart failure; MI: myocardial infarction; AF: atrial fibrillation; IHD: ischaemic heart disease.

We identified 132 923 patients in CPRD-GOLD who met our minimum eligibility criteria. After applying further inclusion criteria, 36 382 patients were included in the final study population (figure 2). The median follow-up time was 3.6 years (IQR 1.7–6.1 years). The median rate of FEV_1_ decline was –19.4 mL·year^−1^ (IQR –40.5–1.9 mL·year^−1^) and thus patients were categorised as having accelerated FEV_1_ decline if they had an FEV_1_ decline faster than –40.5 mL·year^−1^. This meant that 9095 patients (25%) were classed as having accelerated decline and 27 287 (75%) were classed as not having accelerated decline. Patients had a median of three (IQR 2–4) FEV_1_ measurements over the 3-year baseline period. Supplementary figures E1 and E2 illustrate the number of FEV_1_ measurements and time intervals between measurements during the baseline period.

Table 1 reports baseline characteristics for patients with and without accelerated FEV_1_ decline. Patients with accelerated decline were more likely to be male, have severe airflow obstruction (lower FEV_1_ % predicted) and be current smokers, but less likely to have hypertension. Patients were similar in terms of all other CVD characteristics (diabetes and statin use).

**TABLE 1:** Baseline characteristics of patients with and without accelerated FEV_1_ decline

**Baseline characteristics**	**Non-accelerated FEV_1_ decline**	**Accelerated FEV_1_ decline**
**Participants n**	27 287	9095
**Male**	12 942 (47.4)	5381 (59.2)
**Age years**	68.9 (61.7–76.1)	66.8 (59.6–74.0)
**Smoking status**		
Current smokers	16 912 (62.0)	6013 (66.1)
Ex-smokers	10 375 (38.0)	3082 (33.9)
**Airflow obstruction^#^**		
Mild	7566 (27.9)	1567 (17.4)
Moderate	11 771 (43.5)	3851 (42.7)
Severe	6321 (23.3)	2801 (31.0)
Very severe	1424 (5.3)	804 (8.9)
**AECOPD**		
None	10 954 (40.1)	3496 (38.4)
1 moderate, 0 severe	6478 (23.7)	2031 (22.3)
2 moderate, 0 severe	3730 (13.7)	1278 (14.1)
≤3 moderate, 0 severe	4703 (17.2)	1697 (18.7)
1 severe, any moderate	1116 (4.1)	466 (5.1)
≤2 severe, any moderate	306 (1.1)	127 (1.4)
**mMRC score^¶^**		
0	3797 (20.9)	1206 (20.2)
1	7396 (40.6)	2289 (38.4)
2	4448 (24.4)	1526 (25.6)
3	2196 (12.1)	784 (13.1)
4	365 (2.0)	160 (2.7)
**BMI^+^**		
Underweight	1.181 (4.9)	390 (4.9)
Normal	8089 (33.6)	2.750 (34.6)
Overweight	8060 (33.5)	2628 (33.1)
Obese	6727 (28.0)	2180 (27.4)
**Hypertension**	11 770 (43.1)	3660 (40.2)
**Diabetes**	3040 (11.1)	974 (10.7)
**Asthma**	11 238 (41.2)	3566 (39.2)
**Statin use**	8350 (30.6)	2774 (30.5)

Data are presented as n (%) or median (IQR), unless otherwise indicated. FEV_1_: forced expiratory volume in 1 s; AECOPD: acute exacerbation of chronic obstructive pulmonary disease; mMRC: Modified Medical Research Council; BMI: body mass index. ^#^: n=36 105; **^¶^**: n=24 167; **^+^**: n=32 005.

### Risk of CVD

**FIGURE 3:**
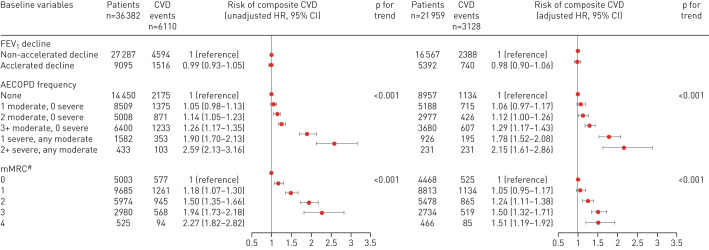
Hazard ratios (95% CI) illustrating the association between accelerated decline in forced expiratory volume in 1 s (FEV_1_), frequency of acute exacerbations of chronic obstructive pulmonary disease (AECOPDs), Modified Medical Research Council (mMRC) score and risk of cardiovascular disease (CVD) outcomes and mortality. ^#^: mMCR score missing in 12 215 patients.

During follow-up, 6110 patients had a CVD event, which equates to a rate of 4.6 events per 100 person-years (95% CI 4.5–4.7 events per 100 person-years). We found no evidence of an association between risk of composite CVD events and accelerated FEV_1_ decline, in either our unadjusted analysis (HR_unadj_ 0.99, 95% CI 0.93–1.05) or in a fully adjusted analysis (HR_adj_ 0.98, 95% CI 0.90–1.06) (figure 3). We did, however, find evidence of an association between increased frequency and severity of AECOPD and breathlessness (mMRC) and CVD outcomes (figure 3). In addition, increasing age, male sex, current smoking, hypertension and statin use all had increased risk for CVD events compared to reference groups (supplementary table E1).

**FIGURE 4:**
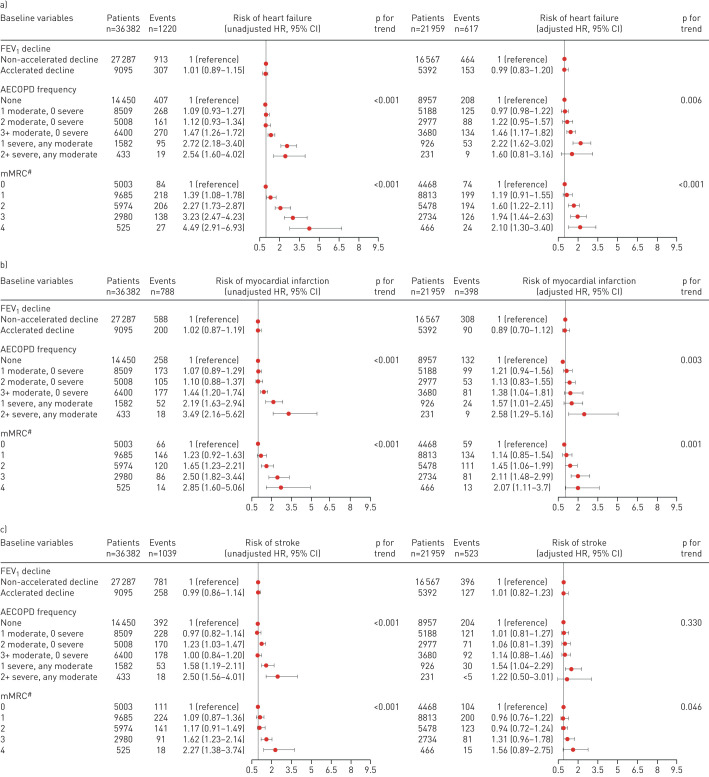

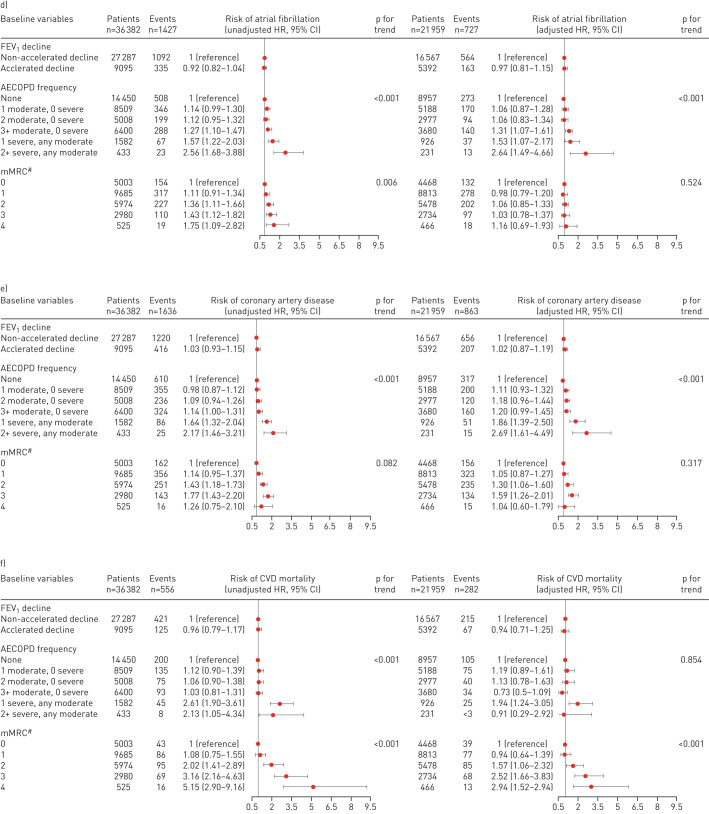
Hazard ratios (95% CI) illustrating the association between accelerated decline in forced expiratory volume in 1 s (FEV_1_), frequency of acute exacerbations of chronic obstructive pulmonary disease (AECOPDs), Modified Medical Research Council (mMRC) score and risk of a) heart failure, b) myocardial infarction, c) stroke, d) atrial fibrillation, e) coronary artery disease and f) cardiovascular disease (CVD) mortality. ^#^: mMCR score missing in proportion of patients under “adjusted” analyses; see table 1 for further information.

Of the 6110 patients who had a CVD event during follow-up, 1220 were recorded as having had heart failure, 788 a myocardial infarction, 1039 a stroke, 1427 atrial fibrillation and 1636 coronary artery disease. There were 556 CVD-related deaths. There was no association between accelerated FEV_1_ decline and heart failure (HR_adj_ 0.99, 95% CI 0.83–1.20), myocardial infarction (HR_adj_ 0.89, 95% CI 0.70–1.12), stroke (HR_adj_ 1.01, 95% CI 0.82–1.23), atrial fibrillation (HR_adj_ 0.97, 95% CI 0.81–1.15), coronary artery disease (HR_adj_ 1.02, 95% CI 0.87–1.19) and CVD mortality (HR_adj_ 0.94, 95% CI 0.71–1.25) (figure 4). Whilst no association was seen between accelerated FEV_1_ decline and risk of individual CVD outcomes, increased frequency and severity of AECOPD and increased mMRC were associated with all CVD outcomes individually in unadjusted models. Supplementary tables E2–E7 provide hazard ratios for all covariates in each model for heart failure, myocardial infarction, stroke, atrial fibrillation and coronary artery disease, respectively.

### Sensitivity analyses

Risk of CVD was similar between patients with and without accelerated decline irrespective of the definitions and cut-offs used to categorise patients according to their rate of loss of lung function (FEV_1_). This finding remained unchanged when accelerated decline was quantified in terms of FEV_1_ % predicted or relative change in FEV_1_ from baseline, and there was no association between the linear rate of FEV_1_ decline and risk of CVD (supplementary table E8). In addition, there was no difference in risk of CVD between patients with FEV_1_ decline in the slowest quartile and all other quartiles (supplementary table E8), between patients with FEV_1_ decline > −20 mL·year^−1^ and patients with FEV_1_ decline in the range −20– −40 mL·year^−1^, −40– −60 mL·year^−1^ and < −60 mL·year^−1^ (supplementary table E9), or between accelerated and non-accelerated FEV_1_ decline using spline regression (supplementary figure E3).

When the analysis was restricted to COPD patients without a history of asthma, there was no difference in CVD risk between patients with and without accelerated FEV_1_ decline (supplementary table E10). Restricting the analysis to events recorded in primary care (GP-diagnosed CVD) and to hospitalisations for CVD did not materially affect our effect estimates (supplementary table E11). No association was seen between accelerated FEV_1_ decline and risk of CVD and mortality after stratification by sex, age, smoking status, AECOPD frequency and airflow obstruction (baseline FEV_1_ % predicted) (supplementary table E12).

Including only those patients who had at least four FEV_1_ measurements or only those with at least two FEV_1_ measurements at least 2 years apart also produced no appreciable change in the hazard ratios for the association between accelerated FEV_1_ decline and CVD risk (supplementary tables E13 and E14). Nor did we observe an association between accelerated FEV_1_ decline and risk of heart failure, myocardial infarction, stroke, atrial fibrillation and coronary artery disease, either as separate outcomes or a composite outcome in the first year of follow-up (supplementary figure E4). Finally, in a sensitivity analysis where we allowed for multiple CVD events during follow-up (rate 9.5 events per 100 person-years, 95% CI 9.2–9.8 events per 100 person-years) we found that the rate of composite CVD, and its individual components, was similar between patients with and without accelerated FEV_1_ decline (supplementary figure E5).

## Discussion

This is the first large observational study to investigate the association between accelerated FEV_1_ decline and risk of CVD outcomes and mortality in COPD. We found that in CVD-naive patients, those with accelerated FEV_1_ decline had a similar risk of CVD outcomes and mortality as patients without accelerated decline, regardless of type of CVD event. Other disease characteristics were more closely related to CVD outcomes and mortality, including history of frequent moderate and severe AECOPD and increased breathlessness.

### Previous studies

No previous studies have investigated the relationship between lung function decline and CVD in a COPD population. A recently published study by Silvestre *et al.* [21] investigated this relationship in a general population of people living in the USA using the ARIC study. The authors found that people with accelerated FEV_1_ decline had a greater risk of CVD compared to those without accelerated decline over a 17-year period. CVD was defined as a composite end point that included hospitalisation or death from heart failure, stroke and coronary heart disease, including myocardial infarction. When each CVD component was analysed separately, they found accelerated FEV_1_ decline was most strongly associated with heart failure and stroke events but not with coronary heart disease events.

Many differences exist between our study and that of Silvestre *et al*. [21] that could explain the differences observed. ARIC is a cohort study that followed participants from 1987 in order to understand the causes of atherosclerosis and other clinical outcomes, including CVD risk factors. Data for these participants were collected systematically at four phases during follow-up by trained healthcare staff. This differs from CPRD-GOLD, a routinely collected healthcare database of information recorded at primary care GP practices across the UK. Whilst there are merits to both types of databases, study results are likely to differ. Furthermore, Silvestre *et al*. [21] used relative change in FEV_1_ % predicted from baseline to estimate accelerated FEV_1_ decline. Our study used absolute change in FEV_1_ adjusting for baseline FEV_1_. Additionally, we performed sensitivity analyses using change in FEV_1_ % predicted and relative change in absolute FEV_1_ and findings were consistent with our main results. Lastly, whilst Silvestre *et al.* [21] adjusted for baseline FEV_1_, other diagnoses such as COPD or airflow obstruction were not accounted for. It is possible that patients with COPD were confounding the relationship between rate of FEV_1_ decline and CVD outcomes. It is also important to note that the magnitude of association with CVD outcomes, between people with and without accelerated FEV_1_, was small in the Silvestre *et al.* study [21], suggesting a marginal increase in their risk (HR_adj_ 1.14, 95% CI 1.06–1.23).

Other studies have found that low lung function (both FEV_1_ and FVC) in early adulthood is associated with risk of CVD in later life in general populations [14, 18–20]. For example, participants enrolled in the ARIC study in the lowest quartile had an increased risk of incident hospitalisation or death from heart failure compared with those in the highest quartile. This risk was also higher in participants with airflow obstruction (FEV_1_/FVC <70%) compared to those without, in both men and women [14]. Again, this is likely driven by COPD patients who generally have a lower FEV_1_ than healthy individuals of the same age. Similarly, the Health, Aging and Body Composition (ABC) study [15] and the Coronary Artery Risk Development in Young Adults (CARDIA) study [16], two US community-based studies, found linear associations between FEV_1_ % predicted and incident heart failure and stroke hospitalisations. Overall, however, there is little evidence to suggest that low lung function, compared to high lung function, in patients with COPD is associated with CVD outcomes and mortality. A recent *post hoc* analysis of SUMMIT trial data found that FEV_1_ % predicted was not associated with CVD events in a COPD population that was at an increased risk of CVD [27]. This is in line with our adjusted findings, for which baseline FEV_1_ % predicted was not associated with risk of CVD and mortality. The association between FEV_1_, FEV_1_ decline and CVD differs between non-COPD general populations and COPD populations.

### Exacerbations of COPD, mMRC score and CVD

Increasing frequency and severity of AECOPD and mMRC score were associated with increased risk of CVD outcomes and mortality in our COPD patient cohort. This suggests that other markers of disease severity, rather than rate of lung function decline, might be more closely related to CVD outcomes and mortality [28]. This observation is in keeping with previous observational studies that have demonstrated that the period immediately following an AECOPD is extremely high risk for CVD events such as myocardial infarction and stroke relative to periods of more stable disease. For example, Rothnie *et al*. [29] used linked UK primary care data (CPRD-GOLD and HES) to investigate the relationship between AECOPD (both moderate and severe) and myocardial infarction and stroke (up to 91 days after an AECOPD). The risk of myocardial infarction and stroke increased after an AECOPD and was higher in patients with more severe AECOPDs. Another observational UK study of primary care patients (THIN database) found that people with COPD had a higher risk of myocardial infarction for up to 5 days after an AECOPD [30]. Likewise, a *post hoc* analysis of data from the SUMMIT study reported an association between AECOPD and increased risk of CVD outcomes (including death, myocardial infarction, stroke, angina and transient ischaemic attack) for up to 1 year after an AECOPD, which was heightened after severe AECOPDs [31].

Few studies have investigated the long-term association between AECOPD frequency and CVD outcomes. One study by Windsor *et al*. [32] used UK primary care GP data (CPRD-GOLD) to perform a case–control study that compared the odds of having a stroke in patients with frequent (two or more AECOPD in the year prior to index date) and infrequent exacerbations. They found no relationship between AECOPD frequency and stroke over a maximum of 9 years [32]. However, this study did not use secondary care data, leading to potentially significant misclassification because they were not able to include more severe AECOPDs or strokes. A previous validation study highlighted the importance of using linked secondary care data with CPRD for identifying events [33]. In particular, the Windsor *et al.* study [32] may well have missed a substantial proportion of more severe AECOPDs or strokes. In contrast, our study, which employed a cohort design in HES and CPRD-GOLD linked data, found that both frequency and severity of AECOPDs were strongly associated with risk of CVD outcomes over a maximum follow-up of 13 years.

### Strengths and limitations

This is the first study to investigate the relationship between rate of lung function decline and risk of CVD outcomes in a COPD population. Using electronic healthcare records, we were able to identify a large population (N=132 923) of COPD patients with varying degrees of disease severity, creating a more generalisable population of COPD patients. We included a wide range of CVD end points (both moderate and severe based on GP-treated or hospitalised CVD events) to capture the varying degrees of CVD event severity. Whilst change in absolute FEV_1_ was our main exposure, following previous studies on lung function decline we included baseline FEV_1_ % predicted as a confounder in our model and performed a sensitivity analysis using relative change in FEV_1_ [2, 8, 34]. Owing to high within-patient variation in absolute FEV_1_, FEV_1_ % predicted is deemed to be better at estimating change in lung function in studies with <5 years of follow-up [35]. We performed a sensitivity analysis using change in FEV_1_ % predicted and results were consistent with those of our main analysis.

To determine the rate of lung function decline at baseline, minimise the effect of measurement error and accurately summarise a patient's lung function decline, based on previous research patients were required to have at least 3 years of baseline follow-up prior to the start of follow-up [21]. The design could therefore have caused immortal time bias. However, baseline trajectories were likely to remain, and it is unlikely that anything disease modifying would have changed a patient's trajectory from accelerated decline to non-accelerated decline, nor would this have influenced the risk of CVD given that no association was found in this study. Another potential issue is that patients with COPD could have been misdiagnosed with asthma and *vice versa*. This is more common in patients over the age of 40 years [36]. This is a limitation of the data because we depend on diagnoses and symptoms recorded by the GP. We performed a sensitivity analysis to attempt to exclude patients who might have been misdiagnosed with COPD. Furthermore, not all patient characteristics or lifestyle factors are recorded within primary and secondary care, *e.g.* physical exercise, so residual confounding is likely. Lastly, statin use was used as a proxy for cholesterol level and pack-years of smoking was not used owing to its poor reliability. Despite this, robust sensitivity analyses were consistent with the main findings.

### Conclusion

We have conducted the first observational study to investigate the relationship between accelerated FEV_1_ decline and risk of CVD outcomes and mortality in COPD patients. We found no association between rate of FEV_1_ decline and composite CVD outcomes or heart failure, myocardial infarction, stroke, atrial fibrillation and coronary artery disease excluding myocardial infarction as separate outcomes. In contrast, frequent and severe AECOPDs and increased breathlessness were associated with risk of CVD outcomes and mortality.

## Supplementary material

10.1183/13993003.00918-2020.Supp1 **Please note:** supplementary material is not edited by the Editorial Office, and is uploaded as it has been supplied by the author.
Supplementary material ERJ-00918-2020.SUPPLEMENT

## Shareable PDF

10.1183/13993003.00918-2020.Shareable1 This one-page PDF can be shared freely online.
Shareable PDF ERJ-00918-2020.Shareable
