# Supplementary material for: Accelerated FEV1 decline and risk of cardiovascular disease and mortality in a primary care population of COPD patients
Source: Eur Respir J. 2021 Mar 4;57(3):2000918. doi: 10.1183/13993003.00918-2020 (PMC7930472; doi:10.1183/13993003.00918-2020)

# Accelerated $FEV_1$ decline and risk of cardiovascular disease and mortality in a primary care population of COPD patients

Supplementary material

## Contents

Table E1: Relationship between accelerated FEV1 decline and risk of composite CVD ........ 1

Table E2: Relationship between accelerated FEV1 decline and risk of heart failure ........ 3

Table E3: Relationship between accelerated FEV1 decline and risk of myocardial infarction ........ 4

Table E4: Relationship between accelerated FEV1 decline and risk of stroke ........ 5

Table E5: Relationship between accelerated FEV1 decline and risk of atrial fibrillation ........ 6

Table E6: Relationship between accelerated FEV1 decline and risk of coronary artery disease excluding myocardial infarction ........ 7

Table E7: Relationship between accelerated FEV1 decline and risk of CVD death ........ 8

Table E8: Relationship between risk of CVD outcomes and accelerated $FEV_1$ decline (by quartiles, measured relative to baseline, measured as percent predicted, and linearly) ........ 9

Table E9: Relationship between rate of FEV1 decline and risk of composite CVD ........ 11

Table E10: Relationship between accelerated $FEV_1$ decline and risk of composite CVD in patients without a history of asthma ........ 12

Table E11: Relationship between accelerated FEV1 decline and risk of GP diagnosed CVD, hospitalised CVD, and death from CVD ........ 13

Table E12: Relationship between accelerated FEV1 decline and risk of composite CVD stratified by gender, age, smoking status, AECOPD, mMRC, and airflow obstruction ........ 14

Table E13: Relationship between accelerated FEV1 decline and risk of composite CVD in patients with at least 4 FEV1 measurements during baseline period ........ 15

Table E14: Relationship between accelerated FEV1 decline and risk of composite CVD in patients with at least 2 FEV1 measurements at least 2 years apart. ........ 16

Figure E1: Number of $FEV_1$ measurements over the three-year baseline period (N=36,382) ........ 18

Figure E2: Time intervals between $FEV_1$ measurements over the three-year baseline period ........ 19

Figure E3: Spline regression with knots at the $10^{th}$, $50^{th}$, and $90^{th}$ percentile ........ 20

Figure E4: Association between composite CVD, heart failure, myocardial infarction, stroke, atrial fibrillation, and coronary artery disease and angina and accelerated $FEV_1$ decline compared to non-accelerated $FEV_1$ decline in the first year of follow-up. ........ 21

Figure E5: Rate of CVD in patients with accelerated FEV1 decline compared to non-accelerated FEV1 decline using multiple CVD events (IRR 95% CI) ........ 22

## Table E1: Relationship between accelerated FEV1 decline and risk of composite CVD

| Exposure and covariates | Unadjusted HR 95% CI | Adjusted HR* (95% CI) |
|---|---|---|
| **Accelerated $FEV_1$ decline** | 0.99 (0.93 – 1.05) | 0.98 (0.90 – 1.06) |
| **AECOPD** | | |
| None | Ref | Ref |
| 1 moderate, 0 severe | 1.05 (0.98 – 1.13) | 1.06 (0.97 – 1.17) |
| 2 moderate, 0 severe | 1.14 (1.05 – 1.23)* | 1.12 (1.00 – 1.26)* |
| ≥3 moderate, 0 severe | 1.26 (1.17 – 1.35)** | 1.29 (1.17 – 1.43)** |
| 1 severe, any moderate | 1.90 (1.70 – 2.13)** | 1.78 (1.52 – 2.08)** |
| ≥2 severe, any moderate | 2.59 (2.13 – 3.16)** | 2.15 (1.61 – 2.86)** |
| **FEV1 % predicted** | | |
| >80% | Ref | Ref |
| 50-80% | 1.05 (0.98 – 1.12) | 1.06 (0.96 – 1.16) |
| 30-50% | 1.15 (1.07 – 1.24)** | 1.02 (0.92 – 1.14) |
| <30% | 1.23 (1.10 – 1.38)** | 1.10 (0.93 – 1.30) |
| **Age** | 1.05 (1.04 – 1.05)** | 1.04 (1.04 – 1.05)** |
| **Men** | 1.33 (1.27 – 1.40)** | 1.40 (1.29 – 1.51)** |
| **Current smokers** | 0.83 (0.78 – 0.87)** | 1.13 (1.05 – 1.22)* |
| **BMI** | | |
| Normal | Ref | Ref |
| Underweight | 1.04 (0.91 – 1.20) | 1.08 (0.89 – 1.30) |
| Overweight | 1.10 (1.03 – 1.18)* | 1.01 (0.93 – 1.10) |
| Obese | 1.11 (1.04 – 1.19)** | 1.09 (0.99 – 1.19) |
| **mMRC** | | |
| 0 | Ref | Ref |
| 1 | 1.18 (1.07 – 1.30)* | 1.05 (0.95 – 1.17) |
| 2 | 1.50 (1.35 – 1.66)** | 1.24 (1.11 – 1.38)** |
| 3 | 1.94 (1.73 – 2.18)** | 1.50 (1.32 – 1.71)** |
| 4 | 2.27 (1.82 – 2.82)** | 1.51 (1.19 – 1.92)* |
| **Asthma** | 0.91 (0.87 – 0.96)** | 1.02 (0.95 – 1.10) |
| **Hypertension** | 1.66 (1.58 – 1.74)** | 1.31 (1.21 – 1.41)** |
| **Diabetes** | 1.39 (1.30 – 1.50)** | 1.09 (0.99 – 1.21) |
| **Statin use** | 1.40 (1.33 – 1.47)** | 1.14 (1.05 – 1.23)* |

*p value <0.05; **p value<0.0001

Table E2: Relationship between accelerated FEV1 decline and risk of heart failure

| Exposure and covariates | Unadjusted HR 95% CI | Adjusted HR* (95% CI) |
|---|---|---|
| **Accelerated $FEV_1$ decline** | 1.01 (0.89 – 1.15) | 0.99 (0.83 – 1.20) |
| **AECOPD** | | |
| None | Ref | Ref |
| 1 moderate, 0 severe | 1.09 (0.93 – 1.27) | 0.97 (0.78 – 1.22) |
| 2 moderate, 0 severe | 1.12 (0.93 – 1.34) | 1.22 (0.95 – 1.57) |
| ≥3 moderate, 0 severe | 1.47 (1.26 – 1.72)** | 1.46 (1.17 – 1.82)* |
| 1 severe, any moderate | 2.72 (2.18 – 3.40)** | 2.22 (1.62 – 3.02)** |
| ≥2 severe, any moderate | 2.54 (1.60 – 4.02)** | 1.60 (0.81 – 3.16) |
| **FEV1 % predicted** | | |
| >80% | Ref | Ref |
| 50-80% | 1.18 (1.00 – 1.39)* | 1.21 (0.97 – 1.52) |
| 30-50% | 1.74 (1.48 – 2.06)** | 1.55 (1.21 – 1.98)* |
| <30% | 2.07 (1.64 – 2.62)** | 1.91 (1.34 – 2.70)** |
| **Age** | 1.06 (1.05 – 1.07)** | 1.10 (1.05 – 1.07)** |
| **Men** | 1.23 (1.10 – 1.38)** | 1.61 (1.36 – 1.91)** |
| **Current smokers** | 0.79 (0.70 – 0.88)** | 1.15 (0.97 – 1.36) |
| **BMI** | | |
| Normal | Ref | Ref |
| Underweight | 1.27 (0.94 – 1.70) | 1.19 (0.80 – 1.78) |
| Overweight | 1.02 (0.87 – 1.18) | 0.89 (0.72 – 1.09) |
| Obese | 1.35 (1.16 – 1.56)** | 1.31 (1.07 – 1.62)* |
| **mMRC** | | |
| 0 | Ref | Ref |
| 1 | 1.39 (1.08 – 1.78)* | 1.19 (0.91 – 1.55) |
| 2 | 2.27 (1.73 – 2.87)** | 1.60 (1.22 – 2.11)* |
| 3 | 3.23 (2.47 – 4.23)** | 1.94 (1.44 – 2.63)** |
| 4 | 4.49 (2.91 – 6.93)** | 2.10 (1.30 – 3.40)* |
| **Asthma** | 0.85 (0.76 – 0.95)* | 0.88 (0.74 – 1.04) |
| **Hypertension** | 2.30 (2.05 – 2.58)** | 1.70 (1.42 – 2.03)** |
| **Diabetes** | 1.78 (1.54 – 2.07)** | 1.25 (1.00-1.56)* |
| **Statin use** | 1.43 (1.27 – 1.60)** | 0.97 (0.81 – 1.16) |

*p value <0.05; **p value<0.0001

Table E3: Relationship between accelerated FEV1 decline and risk of myocardial infarction

| Exposure and covariates | Unadjusted HR 95% CI | Adjusted HR* (95% CI) |
|---|---|---|
| **Accelerated $FEV_1$ decline** | 1.02 (0.87 – 1.19) | 0.89 (0.70 – 1.12) |
| **AECOPD** | | |
| None | Ref | Ref |
| 1 moderate, 0 severe | 1.07 (0.89 – 1.29) | 1.21 (0.94 – 1.56) |
| 2 moderate, 0 severe | 1.10 (0.88 – 1.37) | 1.13 (0.83 – 1.55) |
| ≥3 moderate, 0 severe | 1.44 (1.20- 1.74)** | 1.38 (1.04 – 1.81)* |
| 1 severe, any moderate | 2.19 (1.63 – 2.94)** | 1.57 (1.01 – 2.45)* |
| ≥2 severe, any moderate | 3.49 (2.16 – 5.62)** | 2.58 (1.29 – 5.16)* |
| **FEV1 % predicted** | | |
| >80% | Ref | Ref |
| 50-80% | 1.01 (0.84 – 1.21) | 1.08 (0.83 – 1.40) |
| 30-50% | 1.30 (1.08 – 1.58)* | 1.18 (0.88 – 1.59) |
| <30% | 1.31 (0.97 – 1.78) | 1.08 (0.68 – 1.72) |
| **Age** | 1.04(1.03 – 1.04)** | 0.14 (2.03 -1.05)** |
| **Men** | 1.50 (1.31 -1.72)** | 1.44(1.17 – 1.77)* |
| **Current smokers** | 1.09(0.95 – 1.26) | 1.45 (1.17 – 1.81)* |
| **BMI** | | |
| Normal | Ref | Ref |
| Underweight | 1.36 (0.98 – 1.88) | 1.43 (0.94 – 2.17) |
| Overweight | 0.97 (0.82 – 1.16) | 0.87 (0.69 – 1.10) |
| Obese | 0.76 (0.63 – 0.93)* | 0.69 (0.53- 0.91)* |
| **mMRC** | | |
| 0 | Ref | Ref |
| 1 | 1.23 (0.92- 1.63) | 1.14 (0.85 – 1.54) |
| 2 | 1.65 (1.23-2.21)* | 1.45 (1.06 – 1.99)* |
| 3 | 2.50 (1.82 – 3.44)** | 2.11(1.48 – 2.99)** |
| 4 | 2.85 (1.60 -5.06)** | 2.07 (1.11 – 3.7)* |
| **Asthma** | 0.90 (0.79 – 1.03) | 1.07 (0.88 – 1.31) |
| **Hypertension** | 1.21 (1.06 – 1.39)* | 1.08 (0.88 – 1.34) |
| **Diabetes** | 1.30 (1.06 – 1.60)* | 1.03(1.11 – 3.87)* |
| **Statin use** | 1.01 (0.87 – 1.17) | 1.16 (0.93 -1.44) |

*p value <0.05; **p value<0.0001

Table E4: Relationship between accelerated FEV1 decline and risk of stroke

| Exposure and covariates | Unadjusted HR 95% CI | Adjusted HR* (95% CI) |
|---|---|---|
| **Accelerated $FEV_1$ decline** | 0.99 (0.86- 1.14) | 1.01(0.82- 1.23) |
| **AECOPD** | | |
| None | Ref | Ref |
| 1 moderate, 0 severe | 0.97 (0.82 – 1.14) | 1.01 (0.81- 1.27) |
| 2 moderate, 0 severe | 1.23 (1.03 – 1.47)* | 1.06 (0.81- 1.39) |
| ≥3 moderate, 0 severe | 1.00 (0.84 – 1.20) | 1.14 (0.88 – 1.46) |
| 1 severe, any moderate | 1.58 (1.19 -2.11)* | 1.54 (1.04 – 2.29)* |
| ≥2 severe, any moderate | 2.50 (1.56 – 4.01)** | 1.22 (0.50 – 3.01) |
| **FEV1 % predicted** | | |
| >80% | Ref | Ref |
| 50-80% | 1.16 (0.99 – 1.36) | 1.20 (0.95 – 1.50) |
| 30-50% | 1.07 (0.90 – 1.28) | 1.03 (0.79 – 1.36) |
| <30% | 1.28 (0.97 – 1.68) | 1.26 (0.83-1.91) |
| **Age** | 1.06 (1.05 – 1.06)** | 1.05 (1.04 – 1.06)** |
| **Men** | 1.24 (1.10 – 1.40)* | 1.29 (1.07 – 1.55)* |
| **Current smokers** | 0.87 (0.77 – 0.98)* | 1.24 (1.03 – 1.50)* |
| **BMI** | | |
| Normal | Ref | Ref |
| Underweight | 0.83 (0.59 – 1.17) | 0.85 (0.54 – 1.35) |
| Overweight | 0.97 (0.83-1.12) | 0.97 (0.80 – 1.18) |
| Obese | 0.63 (0.53 – 0.76)** | 0.65 (0.51-0.84)* |
| **mMRC** | | |
| 0 | Ref | Ref |
| 1 | 1.09 (0.87 – 1.36) | 0.96 (0.76 – 1.22) |
| 2 | 1.17 (0.91 – 1.49) | 0.94 (0.72 – 1.24) |
| 3 | 1.62 (1.23 -2.14)* | 1.31 (0.96 – 1.78) |
| 4 | 2.27 (1.38 – 3.74)* | 1.56 (0.89 – 2.75) |
| **Asthma** | 0.77 (0.68 – 0.87)** | 0.82(0.68 – 0.99)* |
| **Hypertension** | 1.37 (1.22 – 1.55)** | 1.00 (0.83 – 1.21) |

| | | |
|---|---|---|
| **Diabetes** | 1.23 (1.03 – 1.48)* | 1.23 (0.94-1.61) |
| **Statin use** | 2.30 (1.14 – 1.47)** | 0.97 (0.80 – 1.19) |

*p value <0.05; **p value<0.0001

Table E5: Relationship between accelerated FEV1 decline and risk of atrial fibrillation

| **Exposure and covariates** | **Unadjusted HR 95% CI** | **Adjusted HR* (95% CI)** |
|---|---|---|
| **Accelerated $FEV_1$ decline** | 0.92(0.82-1.04) | 0.97 (0.81 -1.15) |
| **AECOPD** | | |
| None | Ref | Ref |
| | 1.14 (0.99 – 1.30) | 1.06 (0.87 – 1.28) |
| 1 moderate, 0 severe | 1.12 (0.95 – 1.32) | 1.06 (0.83- 1.34) |
| 2 moderate, 0 severe | 1.27 (1.10-1.47)* | 1.31 (1.07 – 1.61)* |
| ≥3 moderate, 0 severe | 1.57 (1.22 – 2.03)* | 1.53 (1.07 – 2.17)* |

| | | |
|---|---|---|
| 1 severe, any moderate | 2.56 (1.68- 3.88)** | 2.64 (1.49 – 4.66)* |
| ≥2 severe, any moderate | | |
| **FEV1 % predicted** | | |
| >80% | Ref | Ref |
| 50-80% | 1.11 (0.97 – 1.28) | 1.06 (0.88 – 1.28) |
| 30-50% | 1.26 (1.09-1.46)* | 1.09(0.87 – 1.36) |
| <30% | 1.20(0.94 – 1.53) | 1.09 (0.75 -1.60) |
| **Age** | 1.07 (1.06 – 1.07)** | 1.07 (1.06 – 1.-8)** |
| **Men** | 1.39 (1.25 – 1.54)** | 1.44 (1.23- 1.68)** |
| **Current smokers** | 0.61 (0.55 – 0.68)** | 0.91 (0.78 – 1.06) |
| **BMI** | | |
| Normal | Ref | Ref |
| Underweight | 0.95 (0.69 -1.32) | 1.28 (0.84 – 1.93) |
| Overweight | 1.29 (1.12-1.48)** | 1.26 (1.05- 1.52)* |
| Obese | 1.37 (1.18 – 1.58)** | 1.50 (1.23 – 1.83)** |
| **mMRC** | | |
| 0 | Ref | Ref |
| 1 | 1.11 (0.91 -1.34) | 0.98 (0.79 – 1.20) |
| 2 | 1.36 (1.11 – 1.66)* | 1.06(0.85 – 1.33) |
| 3 | 1.43 (1.12 – 1.82)* | 1.03 (0.78 – 1.37) |
| 4 | 1.75 (1.09 – 2.82)* | 1.16 (0.69 – 1.93) |
| **Asthma** | 0.01 (0.82-1.01) | 1.09 (0.94-1.27) |
| **Hypertension** | 1.82 (1.64 – 2.12)** | 1.42 (1.21- 1.67)** |
| **Diabetes** | 1.25 (1.07 -1.46)* | 1.09 (0.88 – 1.35) |
| **Statin use** | 1.25 (1.12- 1.40)** | 0.99 (0.84-1.16) |

*p value <0.05; **p value<0.0001

Table E6: Relationship between accelerated FEV1 decline and risk of coronary artery disease excluding myocardial infarction

| Exposure and covariates | Unadjusted HR 95% CI | Adjusted HR* (95% CI) |
|---|---|---|
| **Accelerated $FEV_1$ decline** | 1.03(0.93-1.15) | 1.02(0.87 – 1.19) |
| **AECOPD** | | |
| None | Ref | Ref |
| 1 moderate, 0 severe | 0.98 (0.87 – 1.12) | 1.11 (0.93 – 1.32) |
| 2 moderate, 0 severe | 1.09 (0.94- 1.26) | 1.18 (0.96 – 1.44) |
| ≥3 moderate, 0 severe | 1.14 (1.00 – 1.31)* | 1.20 (0.99 – 1.45) |
| 1 severe, any moderate | 1.64 (1.32 -2.04)** | 1.86 (1.39 – 2.50)** |
| ≥2 severe, any moderate | 2.17 (1.46 - 3.21)** | 2.69 (1.61 – 4.49)** |
| **FEV1 % predicted** | | |
| >80% | Ref | Ref |
| 50-80% | 0.90 (0.80 – 1.01) | 0.91 (0.77 – 1.07) |
| 30-50% | 0.77 (0.68 – 0.89)** | 0.68 (0.56 – 0.84)** |
| <30% | 0.82 (0.66 – 1.03) | 0.70 (0.50- 0.99)* |
| **Age** | 1.02 (1.02 -1.02)** | 1.02 (0.87 – 1.19) |
| **Men** | 1.28 (1.25 -1.52)** | 1.33 (1.15 – 1.53)* |
| **Current smokers** | 0.97 (0.88- 1.07) | 1.31 (1.04 -1.39)* |
| **BMI** | | |
| Normal | Ref | Ref |
| Underweight | 0.94 (0.71 -1.25) | 0.74 (0.47 – 1.16) |
| Overweight | 1.17 (1.03-1.33)* | 1.02 (0.87 – 1.20) |
| Obese | 1.27 (1.12- 1.45)** | 1.13 (0.95 – 1.35) |
| **mMRC** | | Ref |
| 0 | Ref | 1.05 (0.87 – 1.27) |
| 1 | 1.14 (0.95 – 1.37) | 1.30 (1.06 -1.60)* |
| 2 | 1.43 (1.18 – 1.73)** | 1.59 (1.26 – 2.01)** |
| 3 | 1.77 (1.43 – 2.20)** | 1.04 (0.60 – 1.79) |
| 4 | 1.26 (0.75 – 2.10) | |
| **Asthma** | 1.07 (0.97 – 1.18) | 1.17(1.03 -1.34)* |
| **Hypertension** | 1.61 (1.46 – 1.77)** | 1.32 (1.14 – 1.53)** |
| **Diabetes** | 1.42 (1.24 – 1.63)** | 0.94 (0.78 – 1.15) |
| **Statin use** | 1.80 (1.64 – 1.99)** | 1.60 (1.39 – 1.85)** |

*p value <0.05; **p value<0.0001

Table E7: Relationship between accelerated FEV1 decline and risk of CVD death

| Exposure and covariates | Unadjusted HR 95% CI | Adjusted HR* (95% CI) |
|---|---|---|
| **Accelerated $FEV_1$ decline** | 0.96 (0.79-1.17) | 0.94 (0.71-1.25) |

| | | |
|---|---|---|
| **AECOPD** | | |
| None | Ref | Ref |
| 1 moderate, 0 severe | 1.12 (0.90-1.39) | 1.19 (0.89-1.61) |
| 2 moderate, 0 severe | 1.06 (0.90-1.38) | 1.13 (0.78-1.63) |
| ≥3 moderate, 0 severe | 1.03 (0.81-1.31) | 0.73 (0.50-1.09) |
| 1 severe, any moderate | 2.61 (0.80-1.61)** | 1.94 (1.24-3.05)* |
| ≥2 severe, any moderate | 2.13 (1.90-4.34)* | 0.91 (0.29-2.92) |
| **FEV1 % predicted** | | |
| >80% | Ref | Ref |
| 50-80% | 1.23 (0.97-1.57) | 1.12 (0.81-1.54) |
| 30-50% | 1.87 (1.46-2.40)** | 1.23 (0.86-1.77) |
| <30% | 2.18 (1.53-3.09)** | 1.40 (0.83-2.38) |
| **Age** | 1.06 (1.05-1.08)** | 1.06 (1.04-1.07)** |
| **Men** | 1.50 (1.26-1.78)** | 1.80 (1.40-2.32)** |
| **Current smokers** | 1.08 (0.91-1.29) | 1.62 (1.24-2.10)** |
| **BMI** | | |
| Normal | Ref | Ref |
| Underweight | 1.28 (0.87-1.89) | 1.03 (0.59-1.81) |
| Overweight | 0.73 (0.59-0.92)* | 0.63 (0.47-0.86)* |
| Obese | 0.84 (0.67-1.06) | 0.94 (0.70-1.27) |
| **mMRC** | | |
| 0 | Ref | Ref |
| 1 | 1.08 (0.75-1.55) | 0.94 (0.64-1.39) |
| 2 | 2.02 (1.41-2.89) | 1.57 (1.06-2.32)* |
| 3 | 3.16 (2.16-4.63) | 2.52 (1.66-3.83)** |
| 4 | 5.15 (2.90-9.16) | 2.94 (1.52-5.69)* |
| **Asthma** | 0.78 (0.66-0.93)* | 0.80 (0.62-1.03) |
| **Hypertension** | 1.83 (1.55-2.16)** | 1.49 (1.15-1.93)* |
| **Diabetes** | 1.62 (1.29-2.04)** | 0.90 (0.63-1.29) |
| **Statin use** | 1.27 (1.-7-1.51)* | 1.17 (0.90-1.52) |

*p value <0.05; **p value<0.0001

Table E8: Relationship between risk of CVD outcomes and accelerated $FEV_1$ decline (by quartiles, measured relative to baseline, measured as percent predicted, and linearly)

| Model | Unadjusted HR (95% CI) | Adjusted HR (95% CI) |
|---|---|---|
| **Quartile 1 (slowest decline)** | Ref | Ref |
| **Quartile 2** | 1.05 (0.98 – 1.13) | 0.96 (0.87 – 1.06) |
| **Quartile 3** | 1.06 (0.98 – 1.14) | 0.96 (0.87 – 1.06) |
| **Quartile 4 (accelerated decline)** | 1.02 (0.95– 1.10) | 0.95 (0.85 – 1.05) |
| **Decline in relative $FEV_1$** | 1.06 (1.00 – 1.13) | 1.00 (0.91 – 1.09) |
| **Decline in $FEV_1$ % predicted** | 1.05 (0.98 – 1.13) | 1.00 (0.90 – 1.11) |
| **Linear $FEV_1$ decline** | 1.00 (1.00 – 1.00) | 1.00 (1.00 – 1.00) |

*p value <0.05; **p value<0.0001

Table E9: Relationship between rate of FEV1 decline and risk of composite CVD

| Exposure and covariates | Unadjusted HR 95% CI | Adjusted HR* (95% CI) |
|---|---|---|
| **Decline** | | |
| >-20ml/year | Ref | Ref |
| -20 to -40 ml/year | 1.05 (0.99 – 1.12) | 1.00 (0.91 - 1.09) |
| -40 to -60ml/year | 0.98(0.91 – 1.07) | 0.91 (0.81-1.02) |
| <-60ml | 1.01 (0.93 – 1.09) | 1.02 (0.91 – 1.14) |
| **AECOPD** | | |
| None | Ref | Ref |
| 1 moderate, 0 severe | 1.05 (0.98 – 1.13) | 1.06 (0.97 – 1.17) |
| 2 moderate, 0 severe | 1.14 (1.05 – 1.23)* | 1.12 (1.00 – 1.26)* |
| ≥3 moderate, 0 severe | 1.26 (1.17 – 1.35)** | 1.29 (1.17 – 1.43)** |
| 1 severe, any moderate | 1.90 (1.70 – 2.13)** | 1.78 (1.52 – 2.08)** |
| ≥2 severe, any moderate | 2.59 (2.13 – 3.16)** | 2.15 (1.61 – 2.86)** |
| **FEV1 % predicted** | | |
| >80% | Ref | Ref |
| 50-80% | 1.05 (0.98 – 1.12) | 1.06 (0.96 – 1.16) |
| 30-50% | 1.15 (1.07 – 1.24)** | 1.02 (0.92 – 1.14) |
| <30% | 1.23 (1.10 – 1.38)** | 1.10 (0.92 – 1.30) |
| **Age** | 1.05 (1.04 – 1.05)** | 1.04 (1.04 – 1.05)** |
| **Men** | 1.33 (1.27 – 1.40)** | 1.39 (1.29 – 1.50)** |
| **Current smokers** | 0.83 (0.78 – 0.87)** | 1.13 (1.05 – 1.22)* |
| **BMI** | | |
| Normal | Ref | Ref |
| Underweight | 1.04 (0.91 – 1.20) | 1.08 (0.89 – 1.30) |
| Overweight | 1.10 (1.03 – 1.18)* | 1.01 (0.93 – 1.10) |
| Obese | 1.11 (1.04 – 1.19)** | 1.08 (0.99 – 1.19) |
| **mMRC** | | |
| 0 | Ref | Ref |
| 1 | 1.18 (1.07 – 1.30)* | 1.05 (0.95 – 1.17) |
| 2 | 1.50 (1.35 – 1.66)** | 1.24 (1.10 – 1.38)** |
| 3 | 1.94 (1.73 – 2.18)** | 1.50 (1.32 – 1.71)** |
| 4 | 2.27 (1.82 – 2.82)** | 1.52 (1.19 – 1.92)* |
| **Asthma** | 0.91 (0.87 – 0.96)** | 1.02 (0.95 – 1.10) |
| **Hypertension** | 1.66 (1.58 – 1.74)** | 1.31 (1.21 – 1.41)** |
| **Diabetes** | 1.39 (1.30 – 1.50)** | 1.09 (0.99 – 1.21) |
| **Statin use** | 1.40 (1.33 – 1.47)** | 1.14 (1.05 – 1.23)* |

*p value <0.05; **p value<0.0001

Table E10: Relationship between accelerated $FEV_1$ decline and risk of composite CVD in patients without a history of asthma

| Model | Unadjusted HR (95% CI) | Adjusted HR (95% CI) |
|---|---|---|
| **Accelerated FEV1 decline** | 0.98 (0.90 – 1.07) | 0.96 (0.85 – 1.08) |

*p value <0.05; **p value<0.0001

| Model | Crude HR (95% CI) | Adjusted HR (95% CI) |
|---|---|---|
| **GP diagnosed CVD** | 0.96 (0.89 – 1.04) | 0.93 (0.83 – 1.05) |
| **Hospitalised CVD** | 1.02 (0.90 – 1.16) | 0.96 (0.79 – 1.16) |
| **Death from CVD** | 0.96 (0.70 – 1.17) | 0.94 (0.71 – 1.25) |

Table E11: Relationship between accelerated FEV1 decline and risk of GP diagnosed CVD, hospitalised CVD, and death from CVD

*p value <0.05; **p value<0.0001

Table E12: Relationship between accelerated FEV1 decline and risk of composite CVD stratified by gender, age, smoking status, AECOPD, mMRC, and airflow obstruction

| Model | Crude HR (95% CI) | Adjusted HR (95% CI) |
|---|---|---|
| Males | 0.93 (0.85 – 1.03) | 0.98 (0.85 – 1.12) |
| Females | 0.97 (0.90 – 1.04) | 0.98 (0.88– 1.09) |
| **Stratified by age** | | |
| 35-50 | 0.92 (0.62 – 1.38) | 0.58 (0.30 – 1.12) |
| 50-65 | 1.07 (0.95 – 1.20) | 1.02 (0.86 – 1.21) |
| 65-80 | 1.04 (0.96 – 1.13) | 0.91 (0.81 – 1.02) |
| 80≤ | 1.15 (1.00 – 1.32) | 1.11 (0.92 – 1.35) |
| **Stratified by smoking status** | | |
| Ex-smoker | 0.94 (0.86 – 1.03) | 0.93 (0.81 – 1.07) |
| Current smoker | 1.03 (0.96 – 1.11) | 1.00 (0.89 – 1.11) |
| **Stratified by AECOPD** | | |
| 0, 1, or 2 mod & no severe | 0.98 (0.92 – 1.05) | 0.98 (0.89 – 1.09) |
| 3 mod & no severe or any severe | 0.98 (0.88 – 1.09) | 0.99 (0.84 – 1.16) |
| **Stratified by mMRC** | | |
| 0 | 1.05 (0.86 – 1.27) | 1.02 (0.82 – 1.26) |
| 1 | 1.00 (0.88 – 1.14) | 0.98 (0.85 – 1.12) |
| 2 | 0.94 (0.81 – 1.09) | 0.99 (0.84 – 1.16 |
| 3 | 0.89 (0.73 – 1.08) | 0.95 (0.77 – 1.17) |
| 4 | 0.73 (0.46 – 1.16) | 0.73 (0.44 – 1.21) |
| **Stratified by airflow obstruction** | | |
| ≥80% $FEV_1$ predicted | 1.01 (0.88 - 1.16) | 0.97 (0.83 – 1.12) |
| 50-80% $FEV_1$ predicted | 0.99 (0.90 – 1.08) | 1.02 (0.93 – 1.13) |
| 30-50% $FEV_1$ predicted | 0.92 (0.83 – 1.02) | 0.99 (0.88 – 1.11) |
| ≤30% $FEV_1$ predicted | 1.02 (0.83– 1.26) | 1.08 (0.86 – 1.36) |

*p value <0.05; **p value<0.0001

Table E13: Relationship between accelerated FEV1 decline and risk of composite CVD in patients with at least 4 FEV1 measurements during baseline period

| Exposure and covariates | Unadjusted HR 95% CI | Adjusted HR* (95% CI) |
|---|---|---|
| **Accelerated FEV1 decline** | 1.02 (0.92 – 1.14) | 1.04 (0.90 – 1.21) |
| **AECOPD** | | |
| None | Ref | Ref |
| 1 moderate, 0 severe | 1.11 (0.97 – 1.27) | 1.17 (0.98 – 1.40) |
| 2 moderate, 0 severe | 1.20 (1.03 – 1.40)* | 1.22 (0.99 – 1.50) |
| ≥3 moderate, 0 severe | 1.33 (1.17 – 1.51)** | 1.46 (1.22 – 1.76)** |
| 1 severe, any moderate | 2.12 (1.75 – 2.58)** | 2.07 (1.59 – 2.69)** |
| ≥2 severe, any moderate | 2.41 (1.72 – 3.39)** | 2.17 (1.33 – 3.53)* |
| **FEV1 % predicted** | | |
| >80% | Ref | Ref |
| 50-80% | 1.00 (0.88 – 1.14) | 1.09 (0.92 – 1.30) |
| 30-50% | 1.09 (0.95 – 1.25) | 1.39 (0.85 – 1.28) |
| <30% | 1.00 (0.81 – 1.24) | 1.02 (0.74 – 1.41) |
| **Age** | 1.05 (1.04 – 1.05)** | 1.05 (1.04 – 1.05)** |
| **Men** | 1.51 (1.37 – 1.67)** | 1.65 (1.43 – 1.90)** |
| **Current smokers** | 0.85 (0.77 – 0.93)* | 1.23 (1.07 -1.42)* |
| **BMI** | | |
| Normal | Ref | Ref |
| Underweight | 0.80 (0.60 – 1.07) | 0.91 (0.62 – 1.32) |
| Overweight | 1.01 (0.90 – 1.14) | 0.88 (0.75 – 1.03) |
| Obese | 1.01 (0.88 – 1.15) | 0.97 (0.81 – 1.15) |
| **mMRC** | | |
| 0 | Ref | Ref |
| 1 | 1.14 (0.94 – 1.38) | 1.03 (0.84 – 1.26) |
| 2 | 1.55 (1.27 – 1.88)** | 1.28 (1.03 – 1.58)* |
| 3 | 1.84 (1.48 – 2.29)** | 1.49 (1.18 – 1.89)* |
| 4 | 2.35 (1.58 – 3.50)** | 1.50 (0.97 – 2.31) |
| **Asthma** | 0.90 (0.81 – 0.99)* | 1.01 (0.89 – 1.16) |

| | | |
|---|---|---|
| **Hypertension** | 1.64 (1.49 – 1.81)** | 1.41 (1.22 – 1.62)** |
| **Diabetes** | 1.40 (1.21 – 1.61)** | 1.19 (0.99 – 1.44) |
| **Statin use** | 1.39 (1.26 – 1.53)** | 1.02 (0.88 – 1.18) |

*p value <0.05; **p value<0.0001

Table E14: Relationship between accelerated FEV1 decline and risk of composite CVD in patients with at least 2 FEV1 measurements at least 2 years apart.

| Exposure and covariates | Unadjusted HR 95% CI | Adjusted HR* (95% CI) |
|---|---|---|
| **Accelerated FEV1 decline** | 1.07 (0.95 – 1.20) | 0.95 (0.79 – 1.14) |
| **AECOPD** | | |
| None | Ref | Ref |
| 1 moderate, 0 severe | 1.05 (0.98 – 1.13) | 0.95 (0.78 – 1.15) |
| 2 moderate, 0 severe | 1.14 (1.05 – 1.23)* | 0.96 (0.75 – 1.22) |
| ≥3 moderate, 0 severe | 1.26 (1.17 – 1.35)** | 1.13 (0.91 – 1.39) |
| 1 severe, any moderate | 1.90 (1.70 – 2.13)** | 1.54 (1.11 -2.14)* |
| ≥2 severe, any moderate | 2.59 (2.13 – 3.16)** | 1.63 (0.83 – 3.20) |
| **FEV1 % predicted** | | |
| >80% | Ref | Ref |
| 50-80% | 1.05 (0.98 – 1.12) | 0.94 (0.77 – 1.15) |
| 30-50% | 1.15 (1.07 – 1.24)** | 1.14 (0.91 – 1.43) |
| <30% | 1.23 (1.10 – 1.38)** | 1.33 (0.95 – 1.86) |
| **Age** | 1.05 (1.-4 – 1.05)** | 1.04(1.02 – 1.05)** |
| **Men** | 1.33 (1.27 – 140)** | 1.32 (1.12 – 1.55)* |
| **Current smokers** | 0.82 (0.78 – 0.87)** | 1.07 (0.91 – 1.26) |
| **BMI** | | |
| Normal | Ref | Ref |
| Underweight | 1.04 (0.91 – 1.20) | 1.19 (0.79 – 1.77) |
| Overweight | 1.10 (1.03 – 1.18)* | 1.23 (1.02 – 1.48)* |
| Obese | 1.11 (1.04 – 1.19)* | 1.11 (0.90 – 1.37) |
| **mMRC** | | |
| 0 | Ref | Ref |
| 1 | 1.18 (1.07 – 1.30)* | 1.13 (0.90 – 1.41) |
| 2 | 1.50 (1.35 – 1.66)** | 1.39 (1.10 – 1.76)* |
| 3 | 1.94 (1.72 – 2.18)** | 1.52 (1.15 – 2.01)* |
| 4 | 2.27 (1.82 – 2.82)** | 1.76 (1.08 – 2.85)* |
| **Asthma** | 0.92 (0.87 – 0.96)** | 1.01 (0.86 – 1.18) |

| | | |
|---|---|---|
| **Hypertension** | 1.66 (1.58 – 1.74)** | 1.34 (1.14 – 1.59)** |
| **Diabetes** | 1.39 (1.30 – 1.50)** | 1.06 (0.85 – 1.34) |
| **Statin use** | 1.40 (1.33 – 1.47)** | 1.29 (1.09 – 1.53)* |

*p value <0.05; **p value<0.0001

Figure E1: Number of $FEV_1$ measurements over the three-year baseline period (N=36,382)

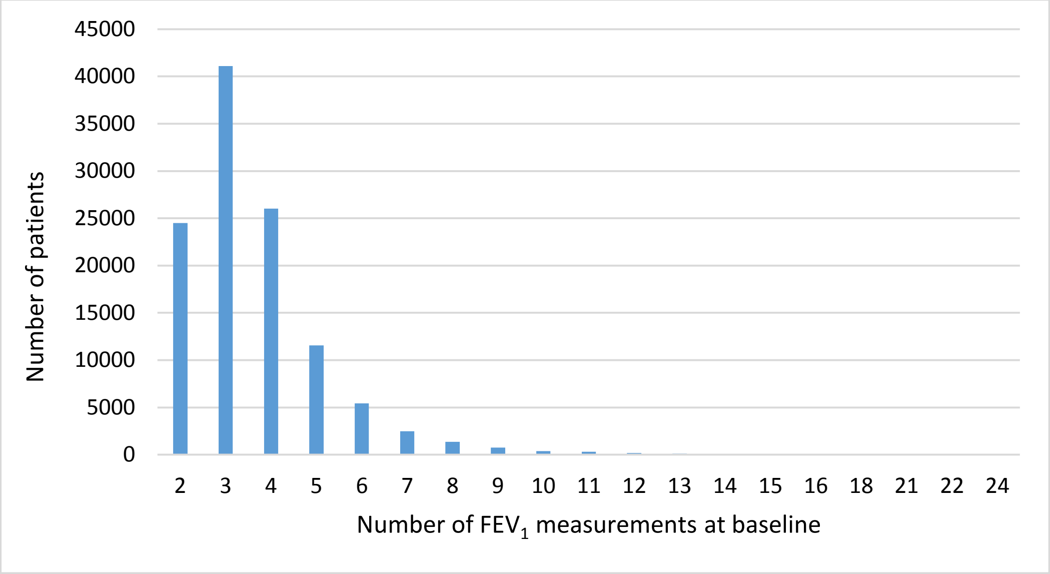

Figure E2: Time intervals between $FEV_1$ measurements over the three-year baseline period

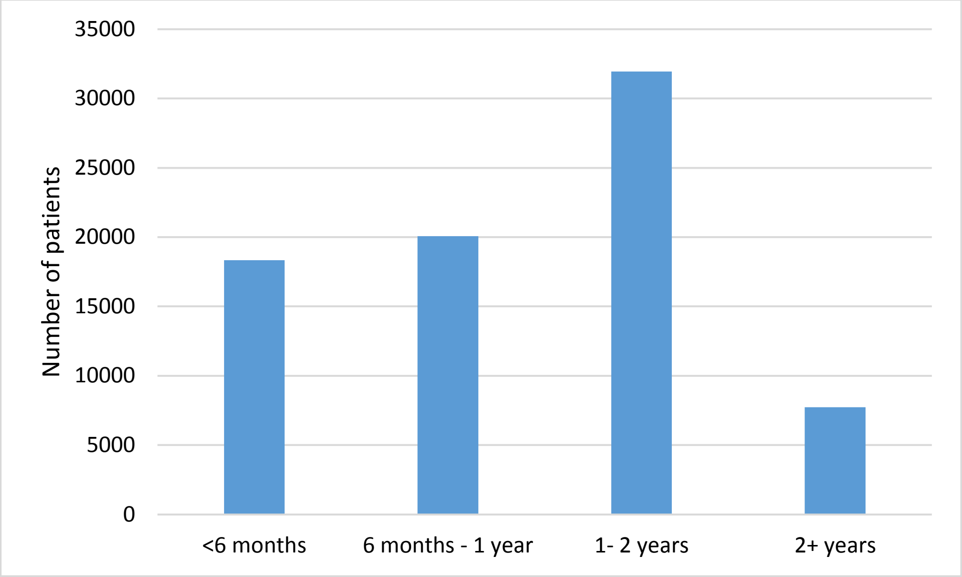

Figure E3: Spline regression with knots at the 10th, 50th, and 90th percentile

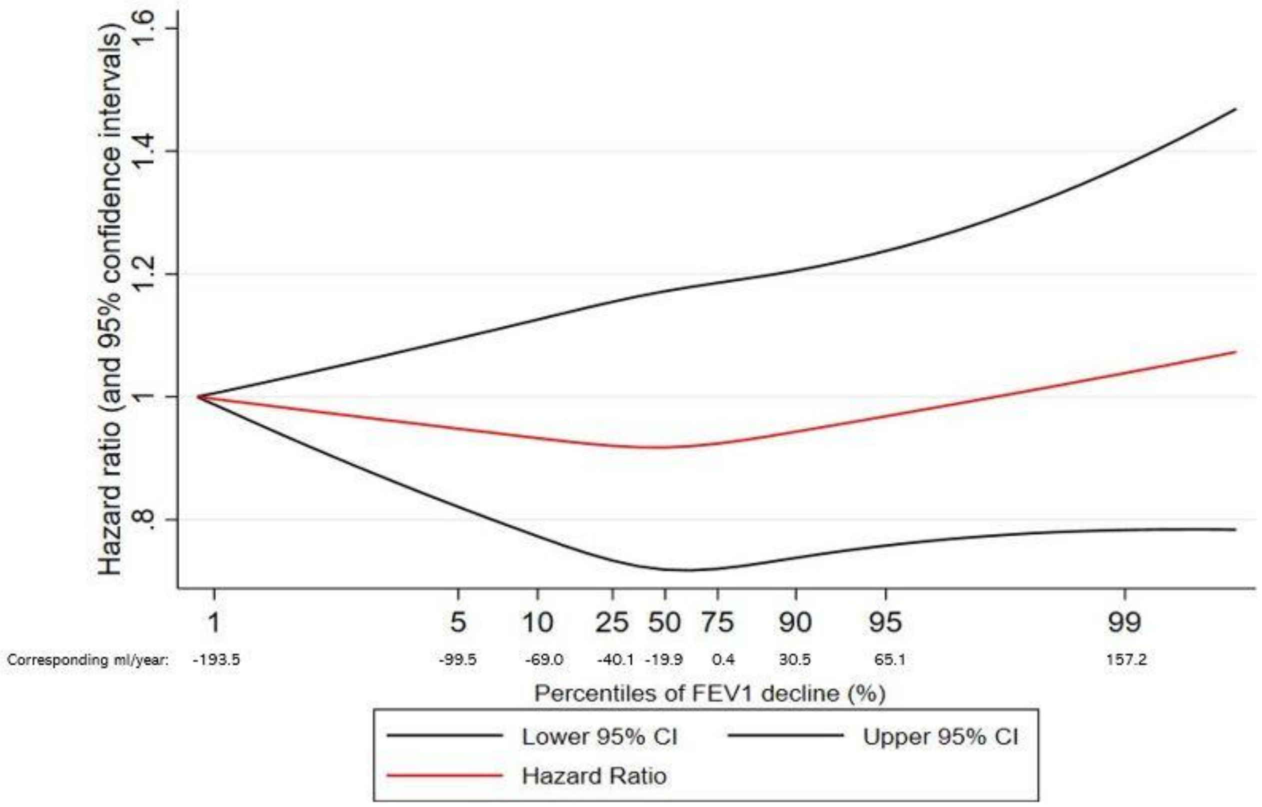

Figure E4: Association between composite CVD, heart failure, myocardial infarction, stroke, atrial fibrillation, and coronary artery disease and angina and accelerated $FEV_1$ decline compared to non-accelerated $FEV_1$ decline in the first year of follow-up.

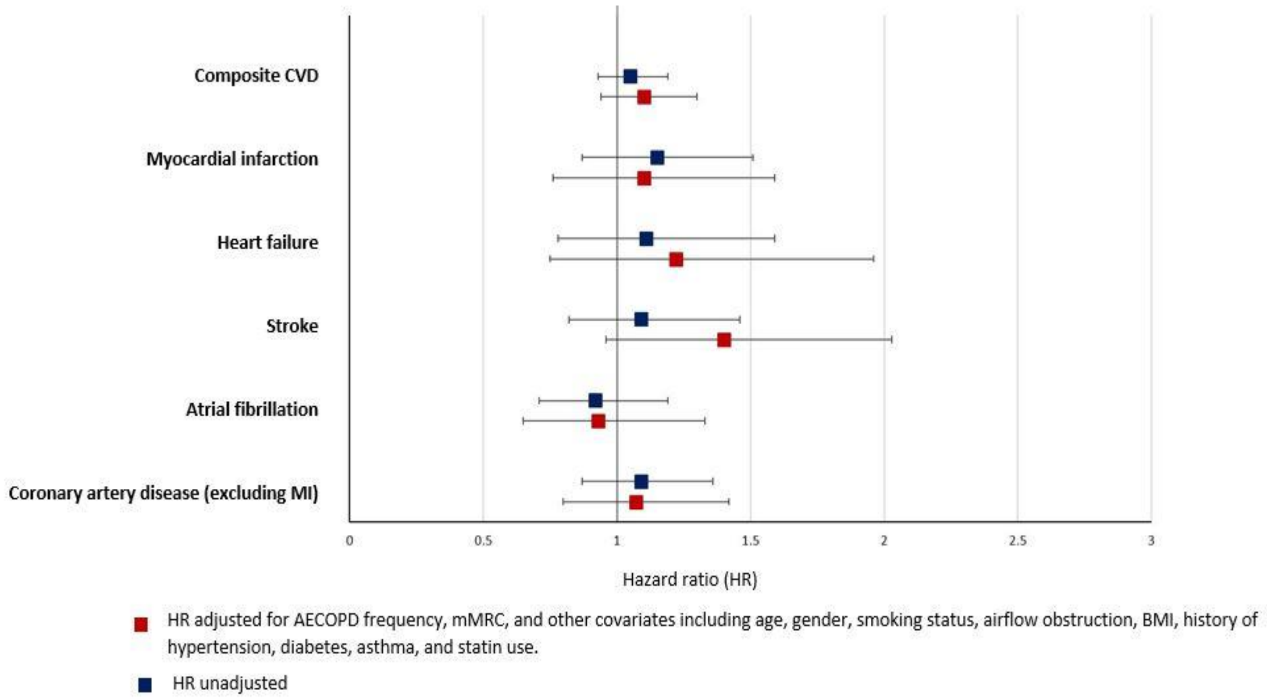

Figure E5: Rate of CVD in patients with accelerated FEV1 decline compared to non-accelerated FEV1 decline using multiple CVD events (IRR 95% CI)

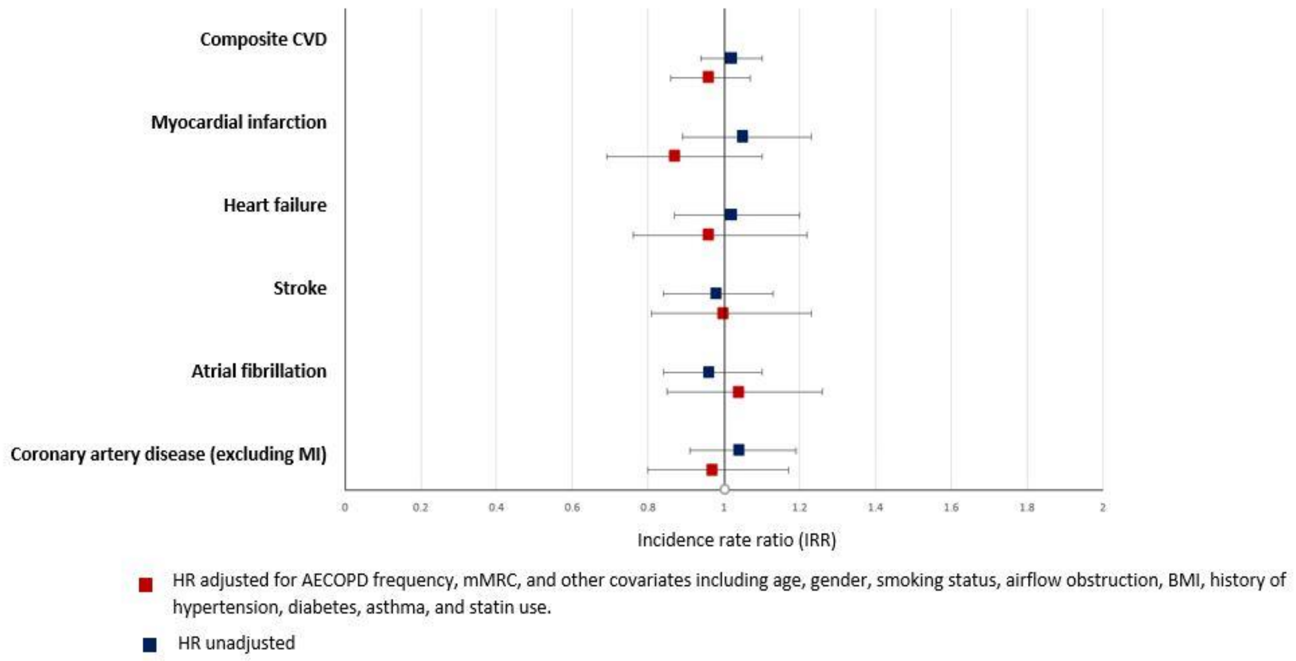

Supplement: Supplementary file 1 [file ERJ-00918-2020.SUPPLEMENT.pdf]
