# Supplementary material for: Accelerated FEV1 decline and risk of cardiovascular disease and mortality in a primary care population of COPD patients
Source: Eur Respir J. 2021 Mar 4;57(3):2000918. doi: 10.1183/13993003.00918-2020 (PMC7930472; doi:10.1183/13993003.00918-2020)
Supplement: Supplementary file 2 [file ERJ-00918-2020.Shareable.pdf]

SHAREABLE PDF

Accelerated FEV_1 decline and risk of cardiovascular disease and mortality in a primary care population of COPD patients

Hannah R. Whittaker[1], Chloe Bloom[1], Ann Morgan[1], Deborah Jarvis[1], Steven J. Kiddle[2,3] and Jennifer K. Quint[1,3]

Affiliations: [1]Respiratory Epidemiology, Occupational Medicine and Public Health, National Heart and Lung Institute, Imperial College London, London, UK. [2]MRC Biostatistics Unit, University of Cambridge, Cambridge, UK. [3]Joint last authors.

Correspondence: Hannah R. Whittaker, Emmanuel Kaye Building, National Heart and Lung Institute, Imperial College, London, UK. E-mail: h.whittaker@imperial.ac.uk

@ERSpublications
In a primary care population of COPD patients, CVD outcomes and mortality were not associated with accelerated FEV_1 decline but with frequent and severe exacerbations of COPD and increased breathlessness https://bit.ly/35APXL6

Cite this article as: Whittaker HR, Bloom C, Morgan A, *et al.* Accelerated FEV_1 decline and risk of cardiovascular disease and mortality in a primary care population of COPD patients. *Eur Respir J* 2021; 57: 2000918 [https://doi.org/10.1183/13993003.00918-2020].

This single-page version can be shared freely online.

ABSTRACT Accelerated lung function decline has been associated with increased risk of cardiovascular disease (CVD) in a general population, but little is known about this association in chronic obstructive pulmonary disease (COPD). We investigated the association between accelerated lung function decline and CVD outcomes and mortality in a primary care COPD population.

COPD patients without a history of CVD were identified in the Clinical Practice Research Datalink (CPRD)-GOLD primary care dataset (n=36 382). Accelerated decline in forced expiratory volume in 1 s (FEV_1) was defined using the fastest quartile of the COPD population's decline. A Cox regression was used to assess the association between baseline accelerated FEV_1 decline and a composite CVD outcome over follow-up (myocardial infarction, ischaemic stroke, heart failure, atrial fibrillation, coronary artery disease and CVD mortality). The model was adjusted for age, sex, smoking status, body mass index, history of asthma, hypertension, diabetes, statin use, Modified Medical Research Council (mMRC) dyspnoea score, exacerbation frequency and baseline FEV_1 % predicted.

6110 COPD patients (16.8%) had a CVD event during follow-up; median length of follow-up was 3.6 years (interquartile range (IQR) 1.7–6.1 years). Median rate of FEV_1 decline was −19.4 $mL \cdot year^{-1}$ (IQR −40.5–1.9); 9095 patients (25%) had accelerated FEV_1 decline (> −40.5 $mL \cdot year^{-1}$), 27 287 (75%) did not (⩽ −40.5 $mL \cdot year^{-1}$). Risk of CVD and mortality was similar between patients with and without accelerated FEV_1 decline (HR_{adj} 0.98, 95% CI 0.90–1.06). Corresponding risk estimates were 0.99 (95% CI 0.83–1.20) for heart failure, 0.89 (95% CI 0.70–1.12) for myocardial infarction, 1.01 (95% CI 0.82–1.23) for stroke, 0.97 (95% CI 0.81–1.15) for atrial fibrillation, 1.02 (95% CI 0.87–1.19) for coronary artery disease and 0.94 (95% CI 0.71–1.25) for CVD mortality. Rather, risk of CVD was associated with a mMRC score ⩾2 and two or more exacerbations in the year prior.

CVD outcomes and mortality were associated with exacerbation frequency and severity and increased mMRC dyspnoea score but not with accelerated FEV_1 decline.

Copyright ©ERS 2021. This version is distributed under the terms of the Creative Commons Attribution Licence 4.0.
